# Supplementary material for: Cellular and Molecular Effects of Eribulin in Preclinical Models of Hematologic Neoplasms
Source: Cancers (Basel). 2022 Dec 10;14(24):6080. doi: 10.3390/cancers14246080 (PMC9776580; doi:10.3390/cancers14246080)
Supplement: Supplementary file 1 [file cancers-14-06080-s001.zip › Vicari et al_Figure S3_R1.pdf]

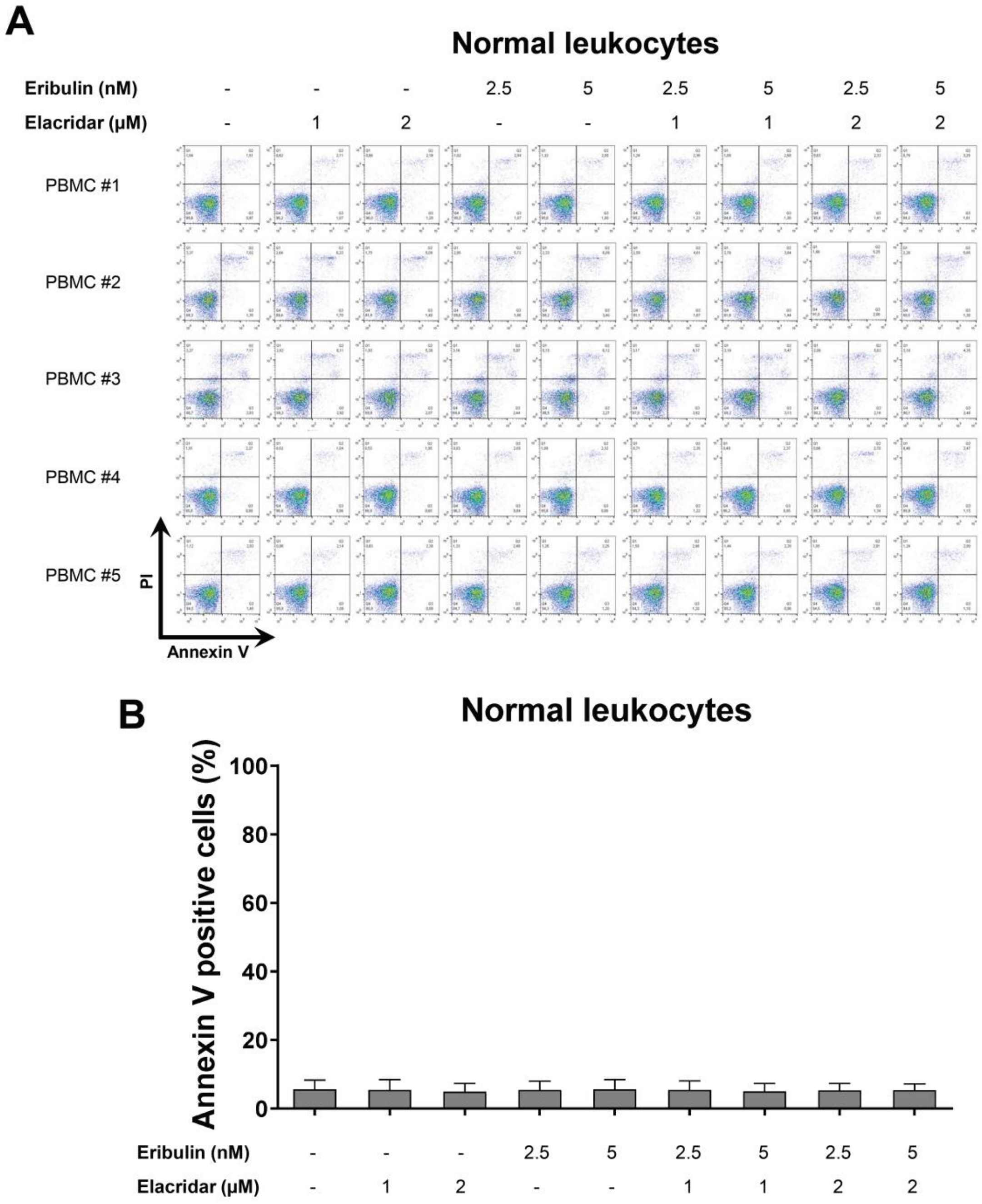

Figure S3. The combination of eribulin and elacridar does not impact the cell viability of normal leukocytes (A) Apoptosis was detected by flow cytometry using APC-annexin V and propidium iodide staining. Representative dot plots are counters for each condition; the upper and lower right quadrants cumulatively the apoptotic population (annexin V+ cells). **(B)** Bar graphs represent the mean ± SD of independent experiments quantifying apoptotic cell death in normal leukocytes (n = 5) after exposure to the vehicle, eribulin (2.5 and 5 nM) and/or elacridar (1 and 2 μM) for 72 hours.
