# Supplementary material for: Cellular and Molecular Effects of Eribulin in Preclinical Models of Hematologic Neoplasms
Source: Cancers (Basel). 2022 Dec 10;14(24):6080. doi: 10.3390/cancers14246080 (PMC9776580; doi:10.3390/cancers14246080)
Supplement: Supplementary file 1 [file cancers-14-06080-s001.zip › Vicari et al_Figure S4_R1.pdf]

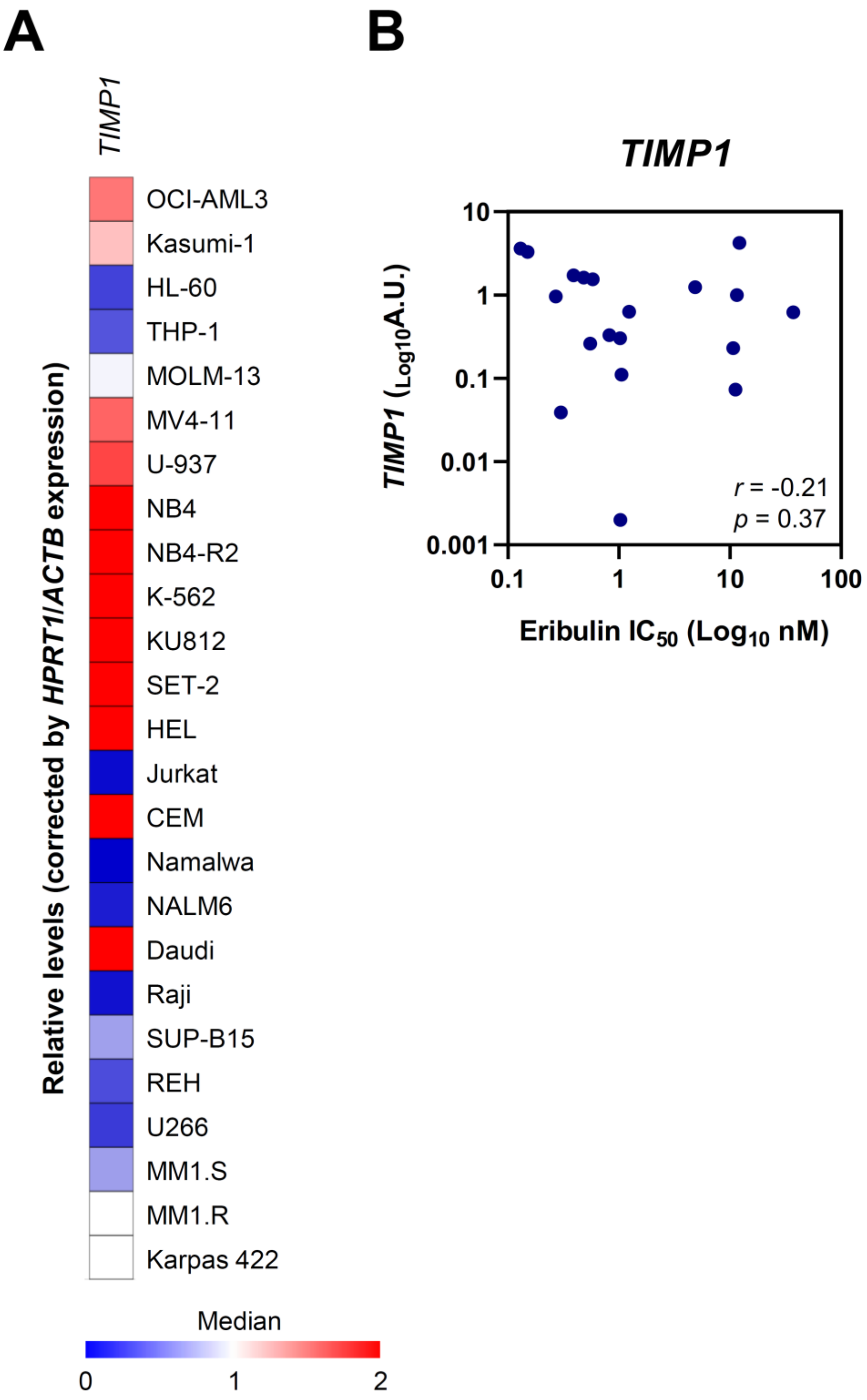

Figure S4. Correlation between *TIMP1* and response to eribulin in blood cancer cells. (A) The heatmap illustrate the expression of TIMP1 in a panel of hematologic neoplasm cell lines. Gene data are represented as relative expression corrected by the expression of *HPRT1/ACTB*, down-regulated and up-regulated genes are given by blue and red, respectively. **(B)** Correlation graph between expression of *TIMP1* and IC50 values for eribulin in blood cancer cells.
