# Supplementary material for: Cellular and Molecular Effects of Eribulin in Preclinical Models of Hematologic Neoplasms
Source: Cancers (Basel). 2022 Dec 10;14(24):6080. doi: 10.3390/cancers14246080 (PMC9776580; doi:10.3390/cancers14246080)
Supplement: Supplementary file 1 [file cancers-14-06080-s001.zip › Vicari et al_Table S1.pdf]

Table S1. Primer sequences and concentrations.

Gene	Sequence	Concentration
ABCB1	FW: GCTCATCGTTTGTCTACAGTTCGT<br>RV: ACAATGACTCCATCATCGAAACC	300 nM
ABCC1	FW: CGACATGACCGAGGCTACATT<br>RV: AGCAGACGATCCACAGCAAAA	300 nM
TUBB3	FW: AGTCGCCCACGTAGTTGC<br>RV: CGCCCAGTATGAGGGAGAT	300 nM
STMN1	FW: AGCCCTCGGTCAAAAGAATC<br>RV: TTCAAGACCTCAGCTTCATGGG	300 nM
TIMP1	FW: ATGGACTCTTGCACATCACTAC<br>RV: GGGATGGATAAACAGGGAAACA	300 nM
ACTB	FW: AGGCCAACCGCGAGAAG<br>RV: ACAGCCTGGATAGCAACGTACA	300 nM
HPRT1	FW: GAACGTCTTGCTCGAGATGTGA<br>RV: TCCAGCAGGTCAGCAAAGAAT	300 nM
