# Supplementary material for: Cellular and Molecular Effects of Eribulin in Preclinical Models of Hematologic Neoplasms
Source: Cancers (Basel). 2022 Dec 10;14(24):6080. doi: 10.3390/cancers14246080 (PMC9776580; doi:10.3390/cancers14246080)
Supplement: Supplementary file 1 [file cancers-14-06080-s001.zip › Vicari et al_Table S2.pdf]

Table S2. Cell lines' characteristics and their sensitivity to eribulin.

Cell line	Hematologic neoplasms	Eribulin IC_{50} (nM)
OCI-AML3	Acute myeloid leukemia	0.58
Kasumi-1	Acute myeloid leukemia	4.86
HL-60	Acute myeloid leukemia	0.55
THP-1	Acute myeloid leukemia	0.82
MOLM-13	Acute myeloid leukemia	0.27
MV4-11	Acute myeloid leukemia	0.48
U-937	Acute myeloid leukemia	0.39
NB4	Acute promyelocytic leukemia	0.15
NB4-R2	Acute promyelocytic leukemia[1]	0.13
K-562	Chronic myeloid leukemia	>100
KU812	Chronic myeloid leukemia	12.12
SET-2	Myeloproliferative neoplasm	>100
HEL	Myeloproliferative neoplasm	>100
Jurkat	T acute lympboblastic leukemia	0.30
CEM	T acute lympboblastic leukemia	>100
Namalwa	Burkitt lymphoma	0.88
NALM6	B acute lymphoblastic leukemia	1.05
Daudi	Burkitts lymphoma	>100
Raji	Burkitts lymphoma	11.17
SUP-B15	B acute lymphoblastic leukemia	1.23
REH	Acute lymphocytic leukemia, non-T; non-B	1.02
U266	Multiple myeloma	10.66
MM1.S	Multiple myeloma	37.03
MM1.R[2]	Multiple myeloma[2]	11.55
Karpas 442	Diffuse large B-cell lymphoma	1.03

[1] Acute promyelocytic leukemia resistant to all transretinoic acid.

[2] Multiple myeloma resistant to glucocorticoids.
