# Supplementary figures and images for: Cellular and Molecular Effects of Eribulin in Preclinical Models of Hematologic Neoplasms
Source: Cancers (Basel). 2022 Dec 10;14(24):6080. doi: 10.3390/cancers14246080 (PMC9776580; doi:10.3390/cancers14246080)

EXPERIMENT #1

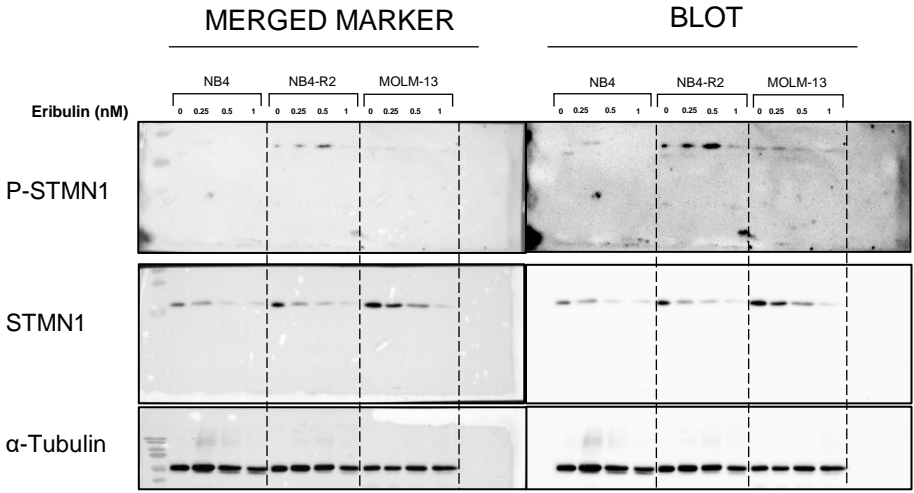

EXPERIMENT #2

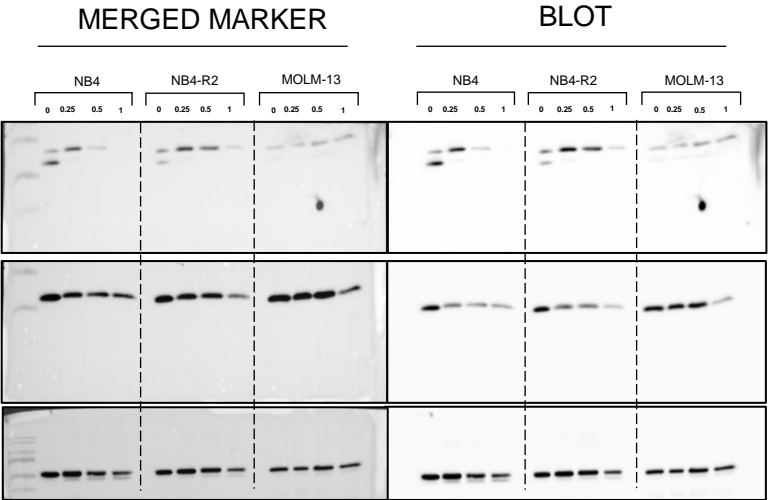

EXPERIMENT #3

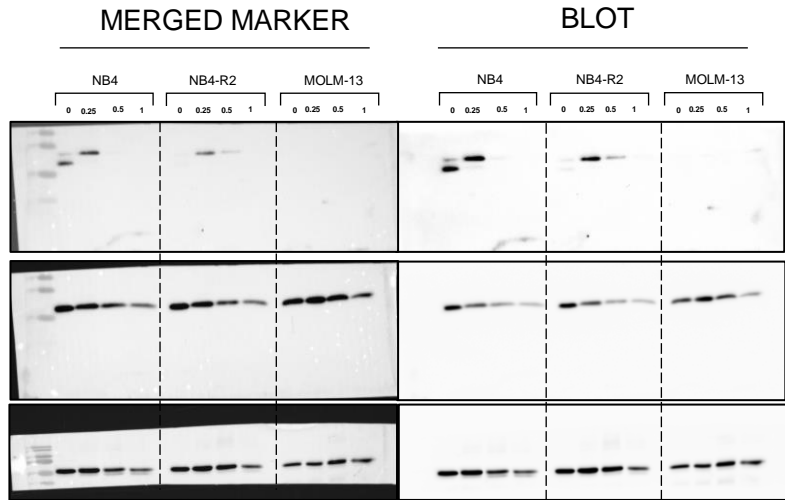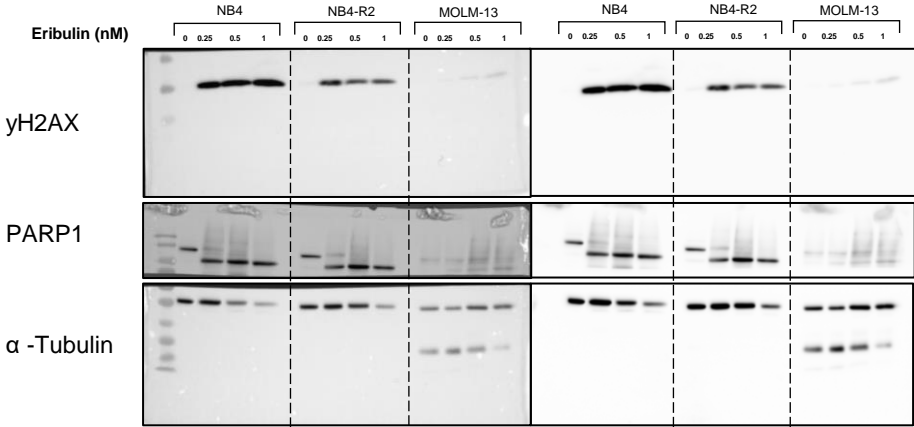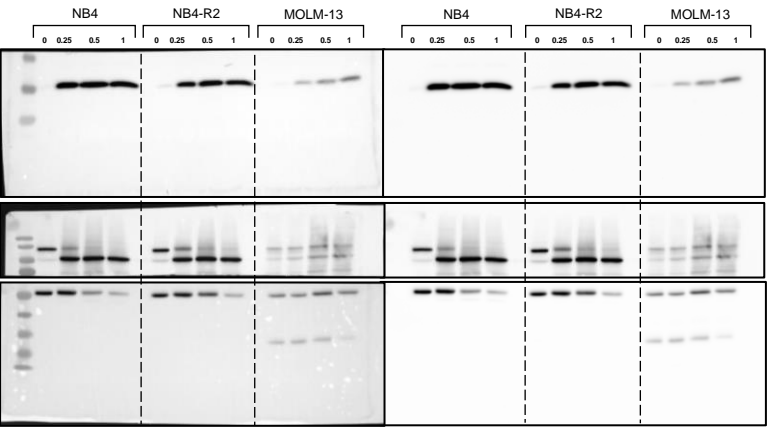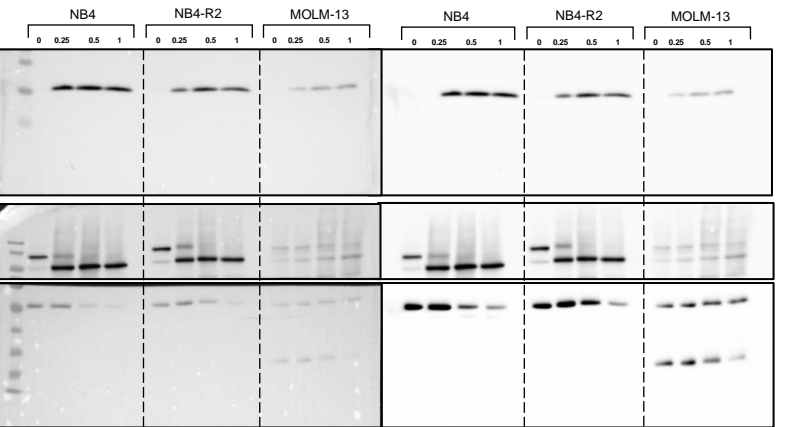

EXPERIMENT #1

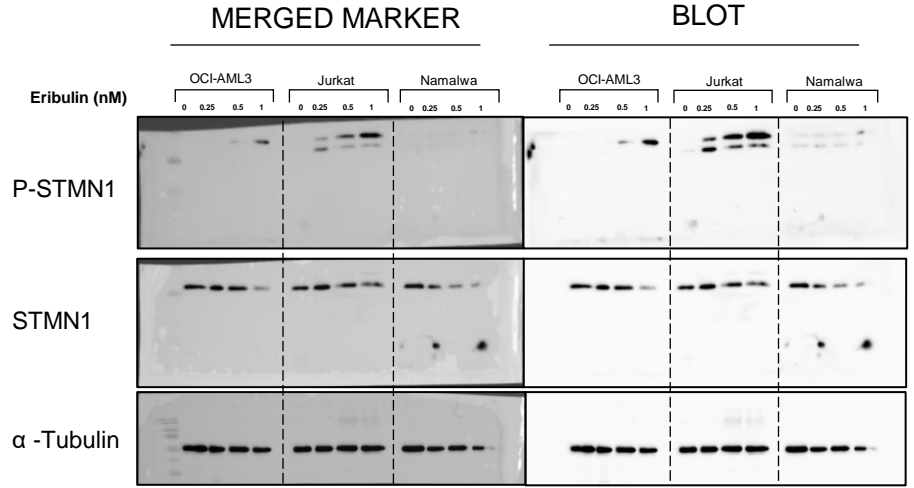

EXPERIMENT #2

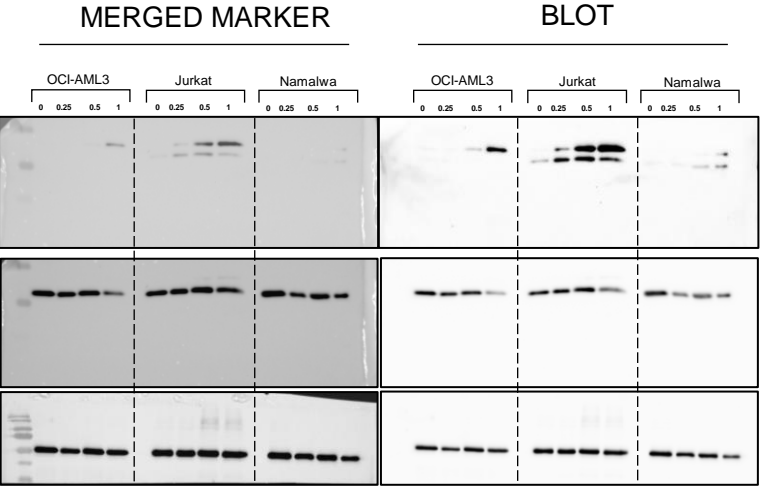

EXPERIMENT #3

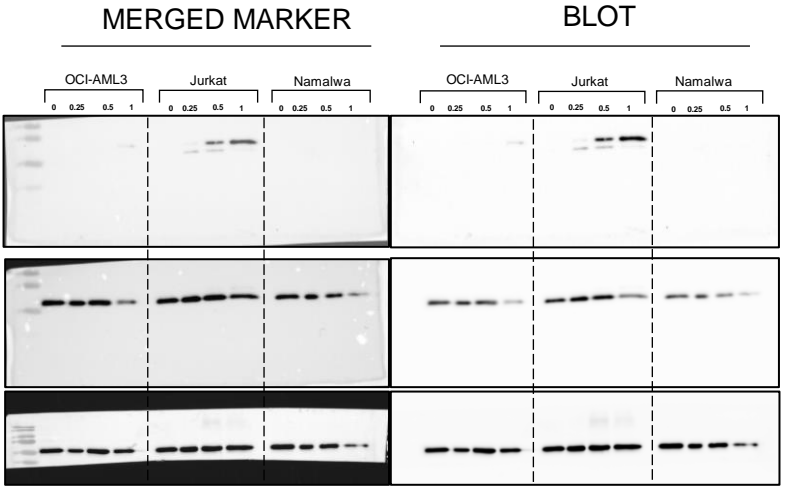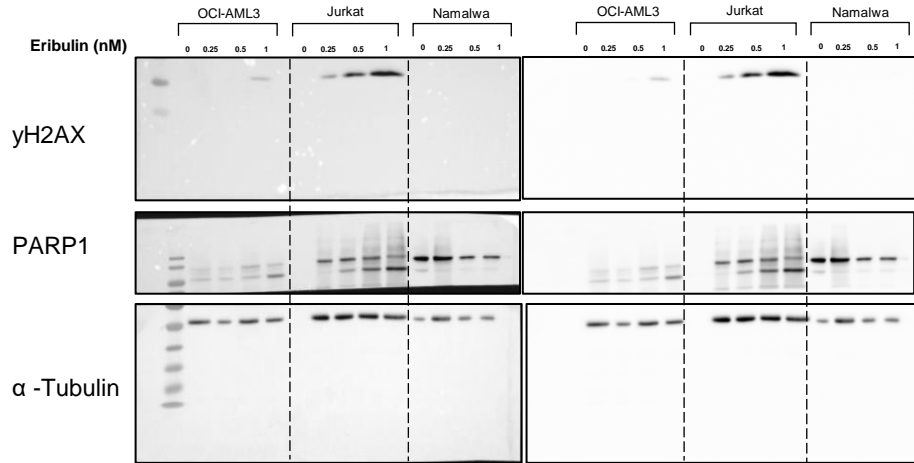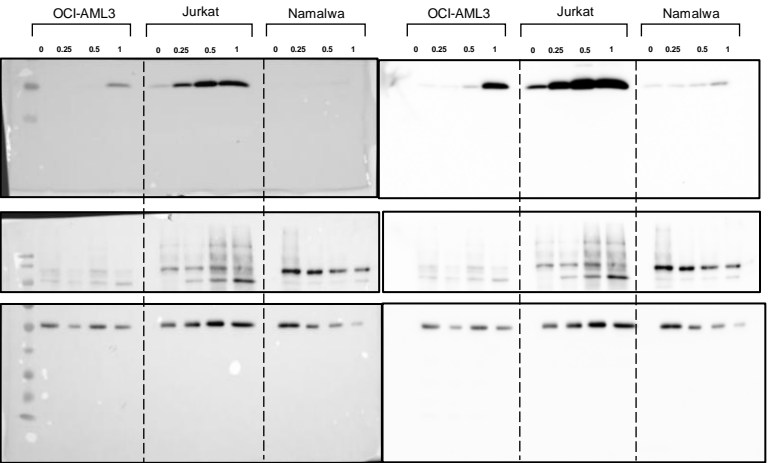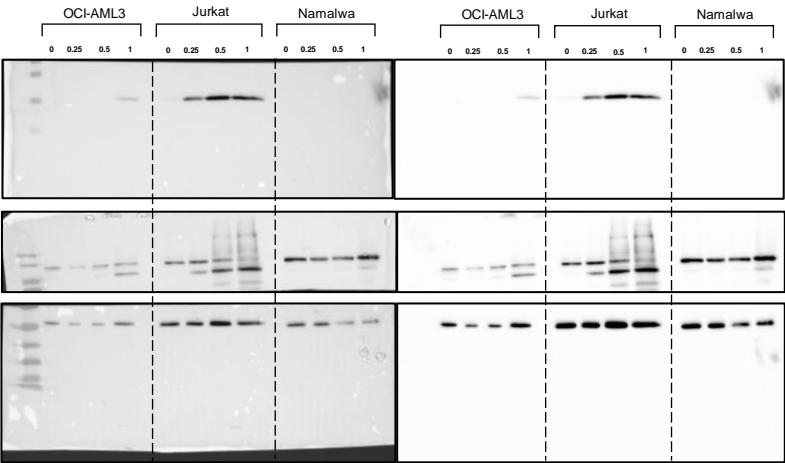

Supplement: Supplementary file 1 [file cancers-14-06080-s001.zip › Vicari et al_Figure S1.pdf]

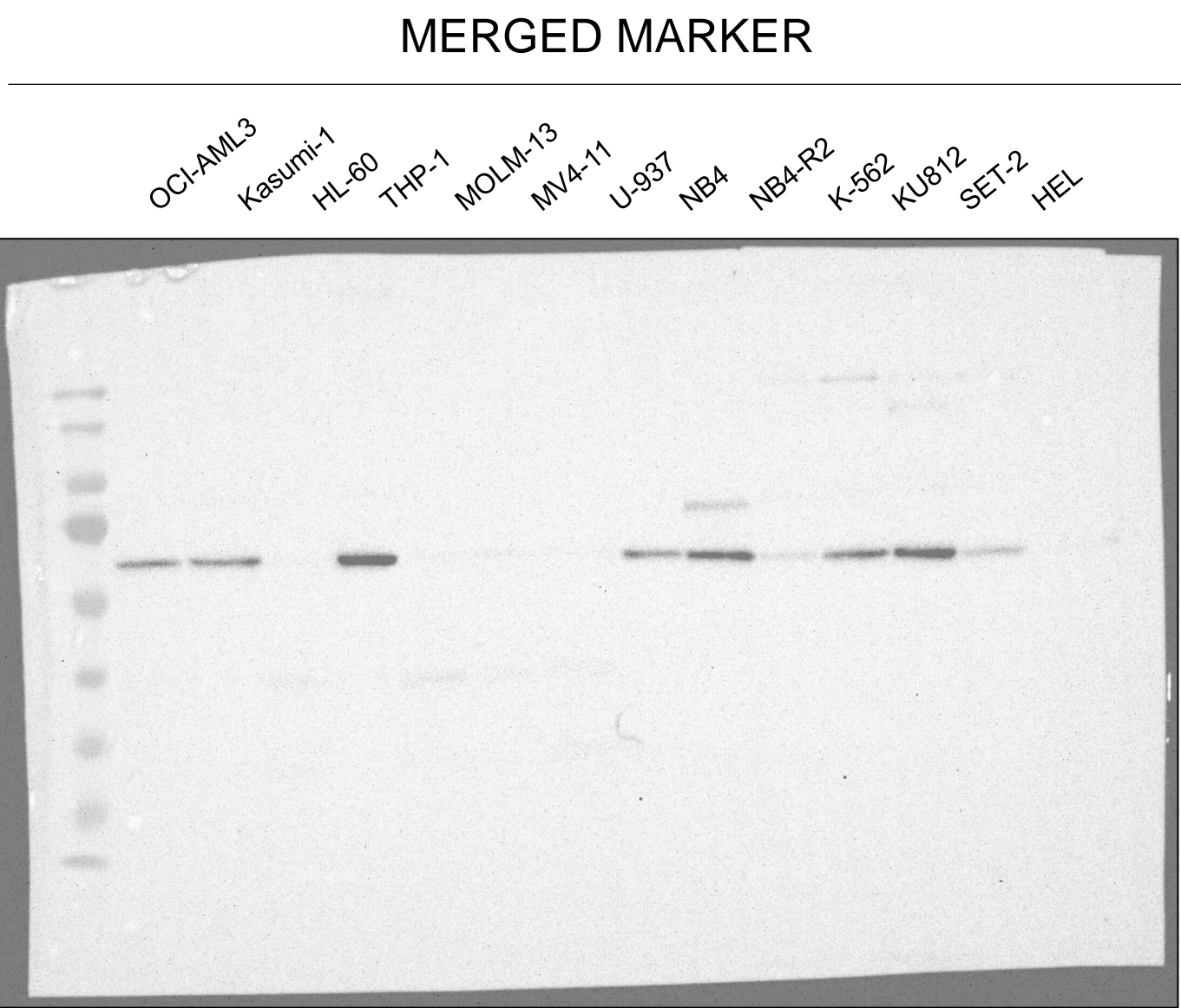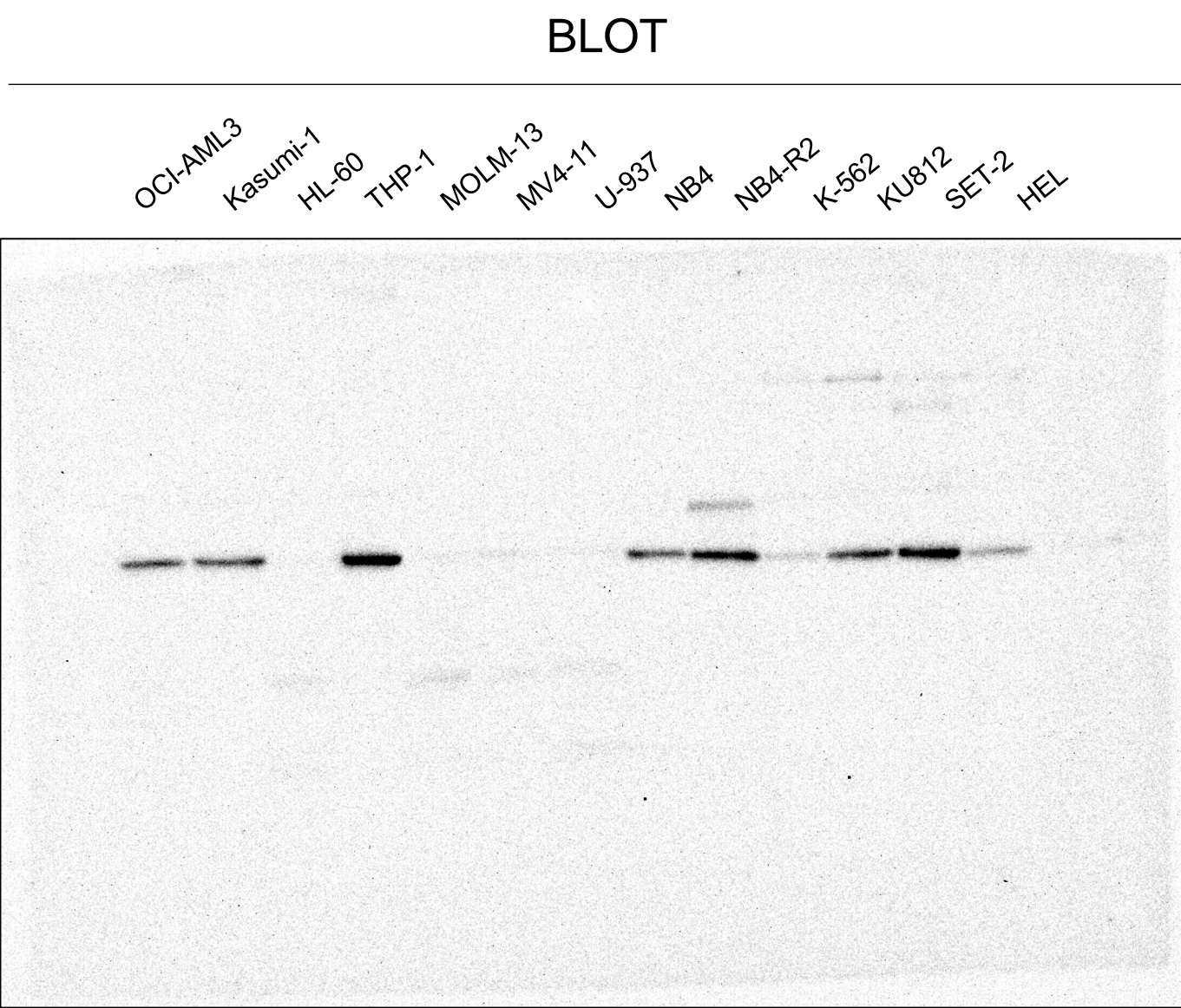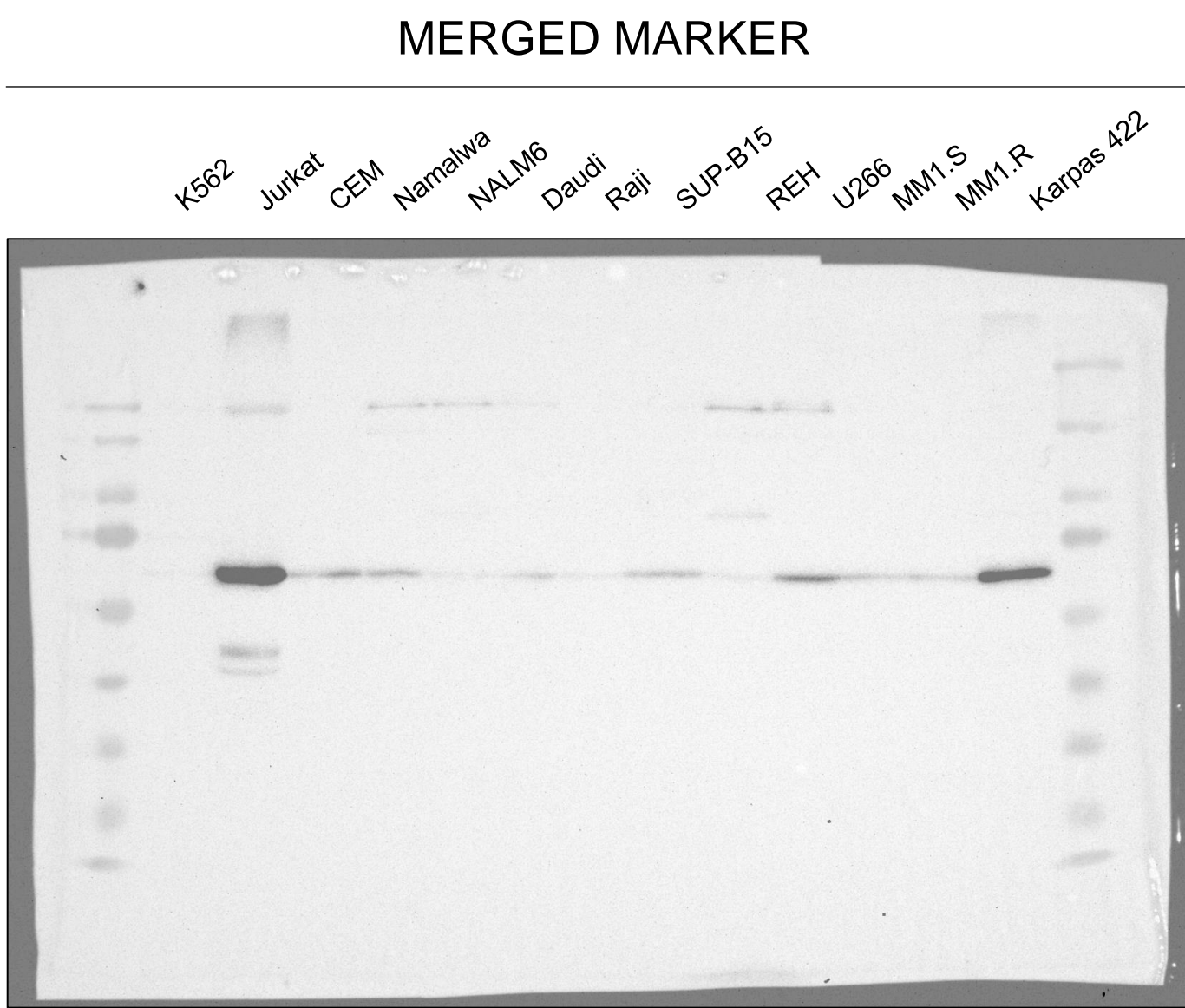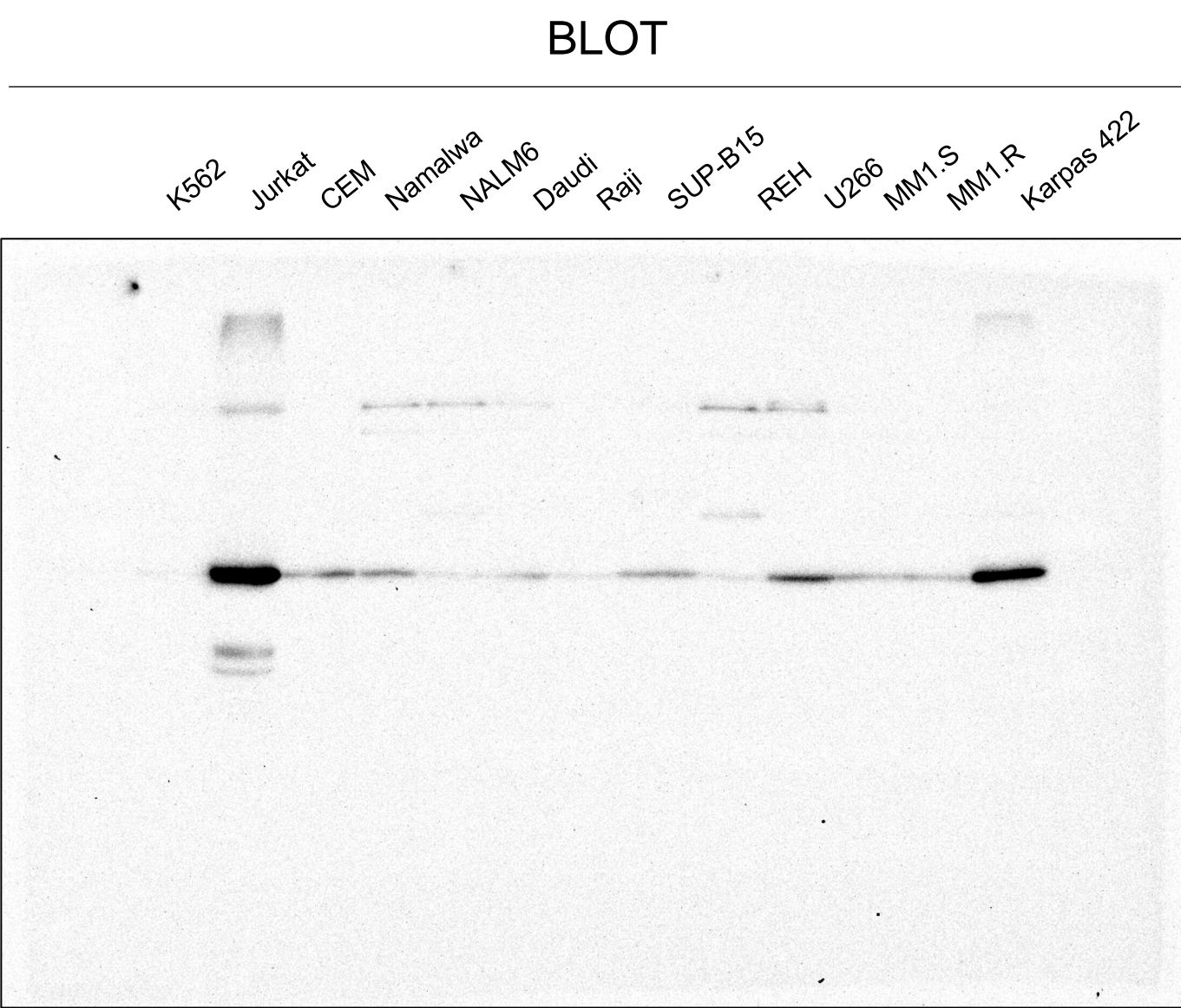

**IB: p-AKT<sup>S473</sup>**  
60 kDa

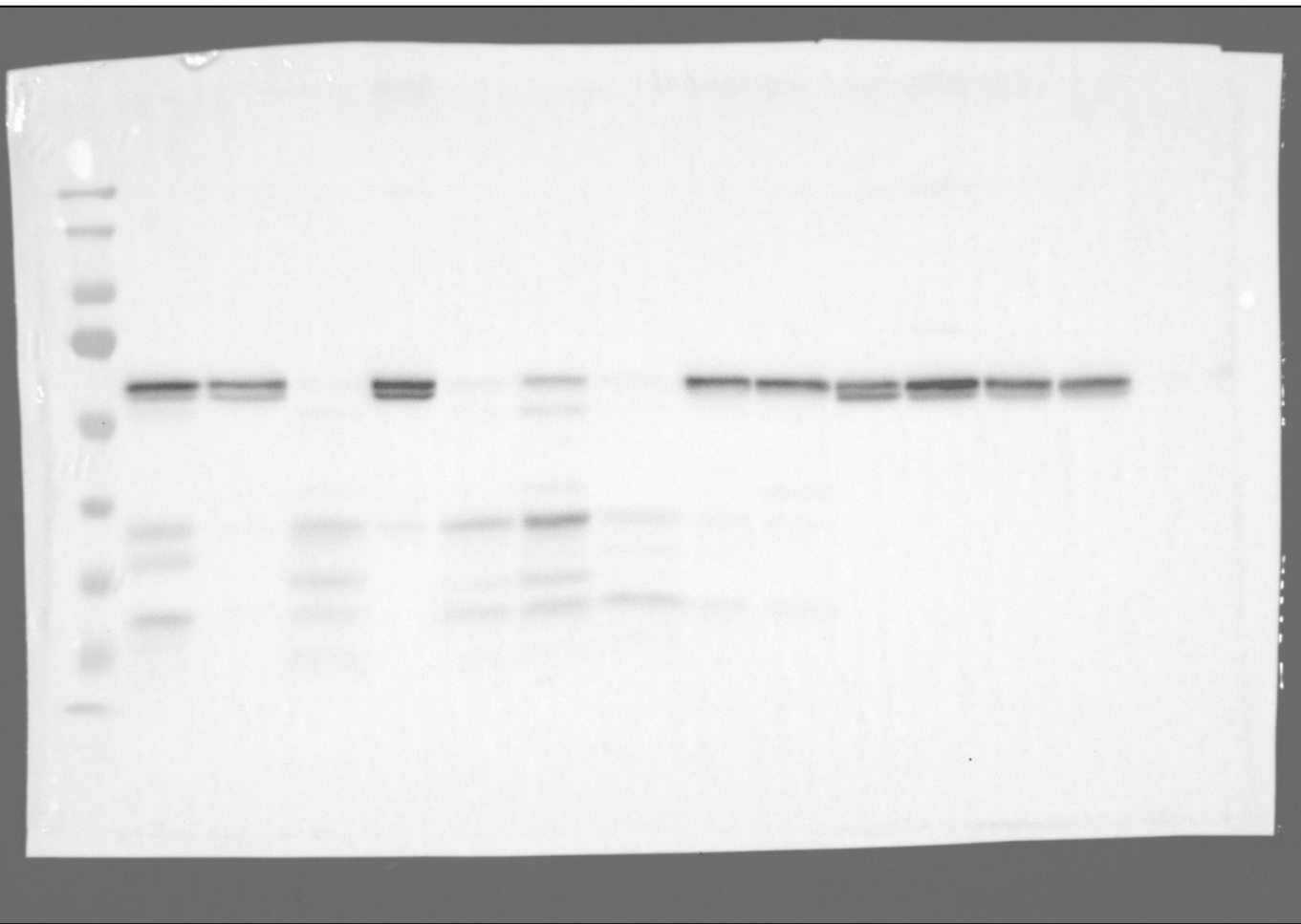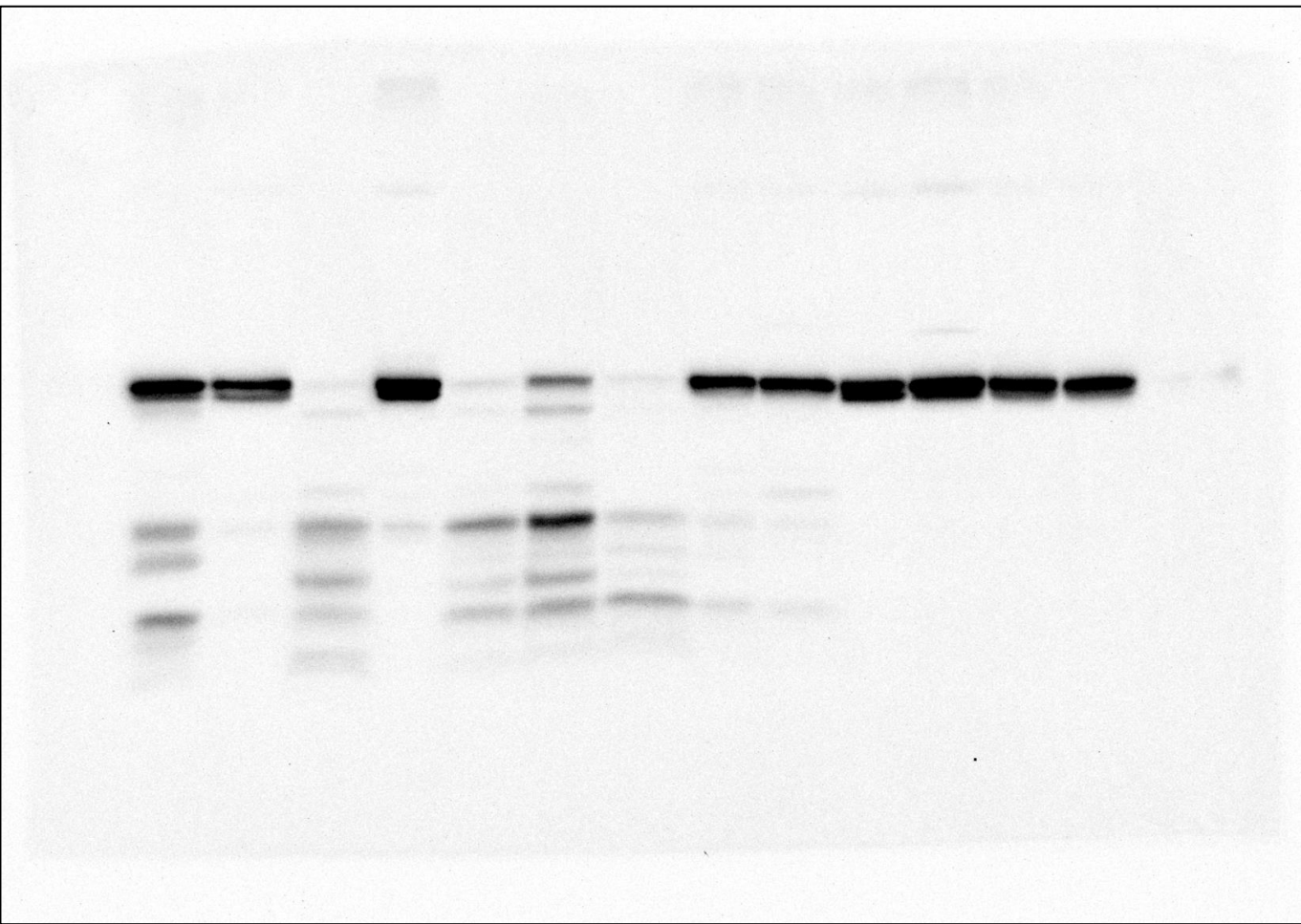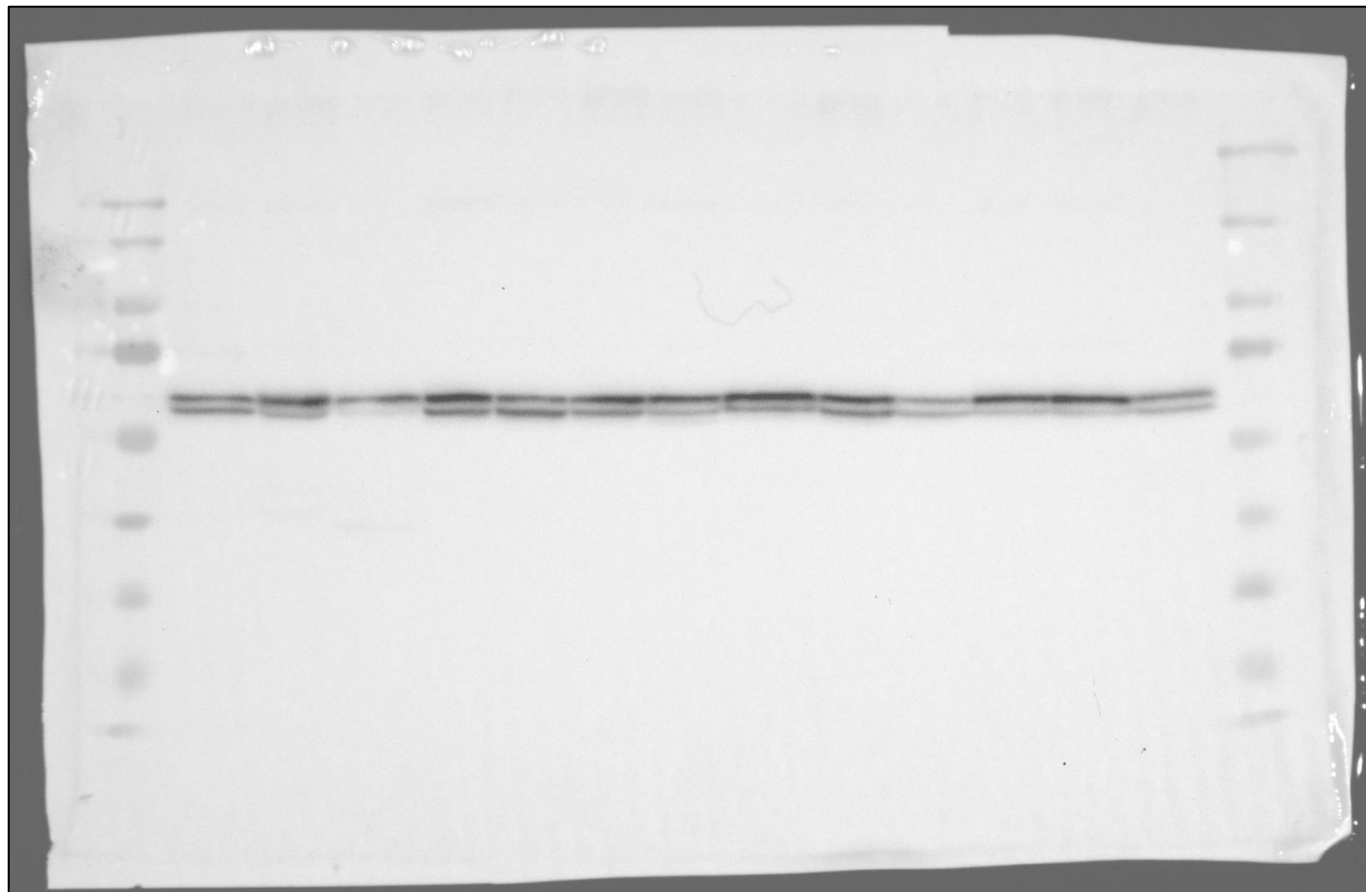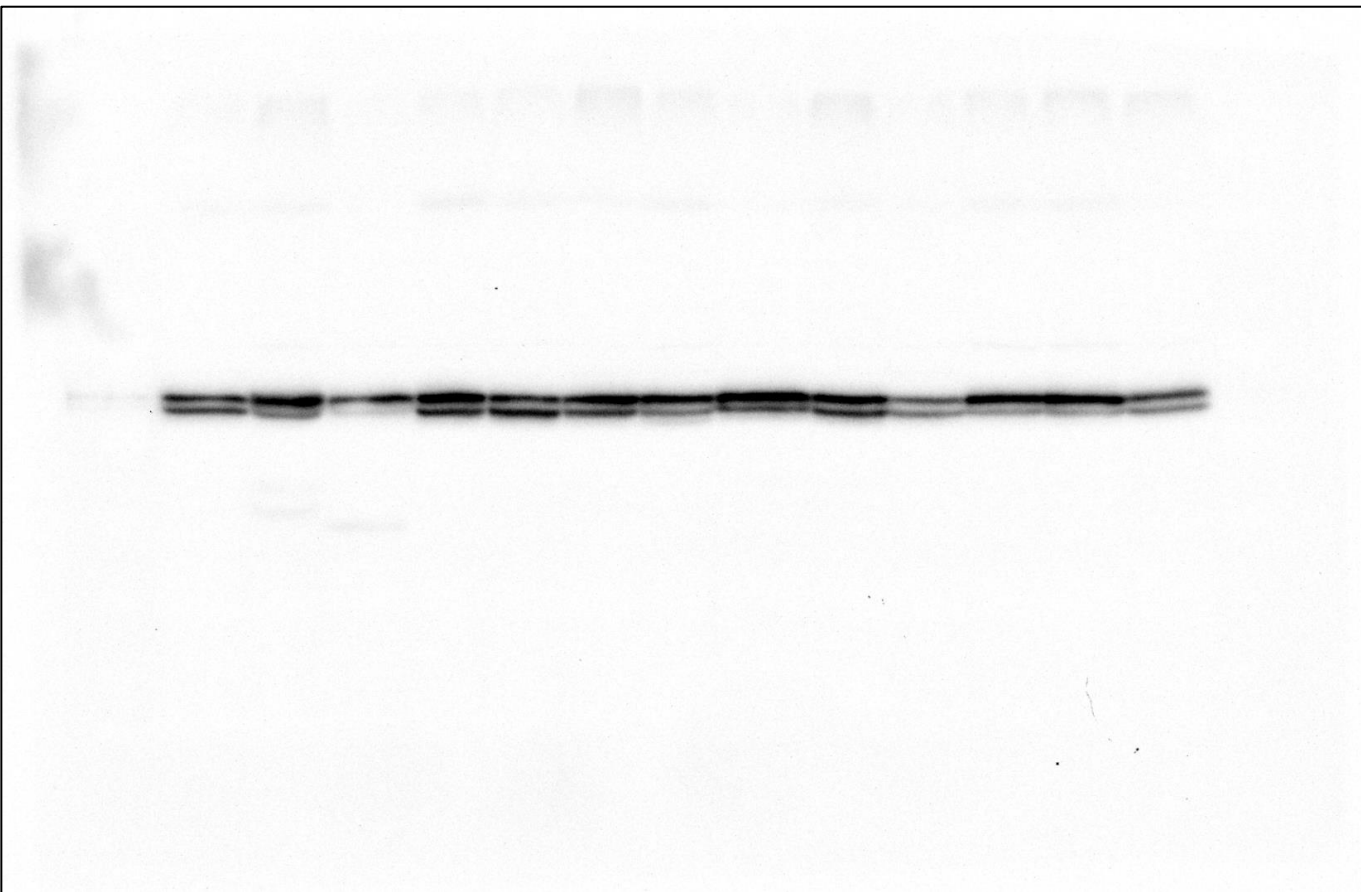

**IB: AKT**  
60 kDa

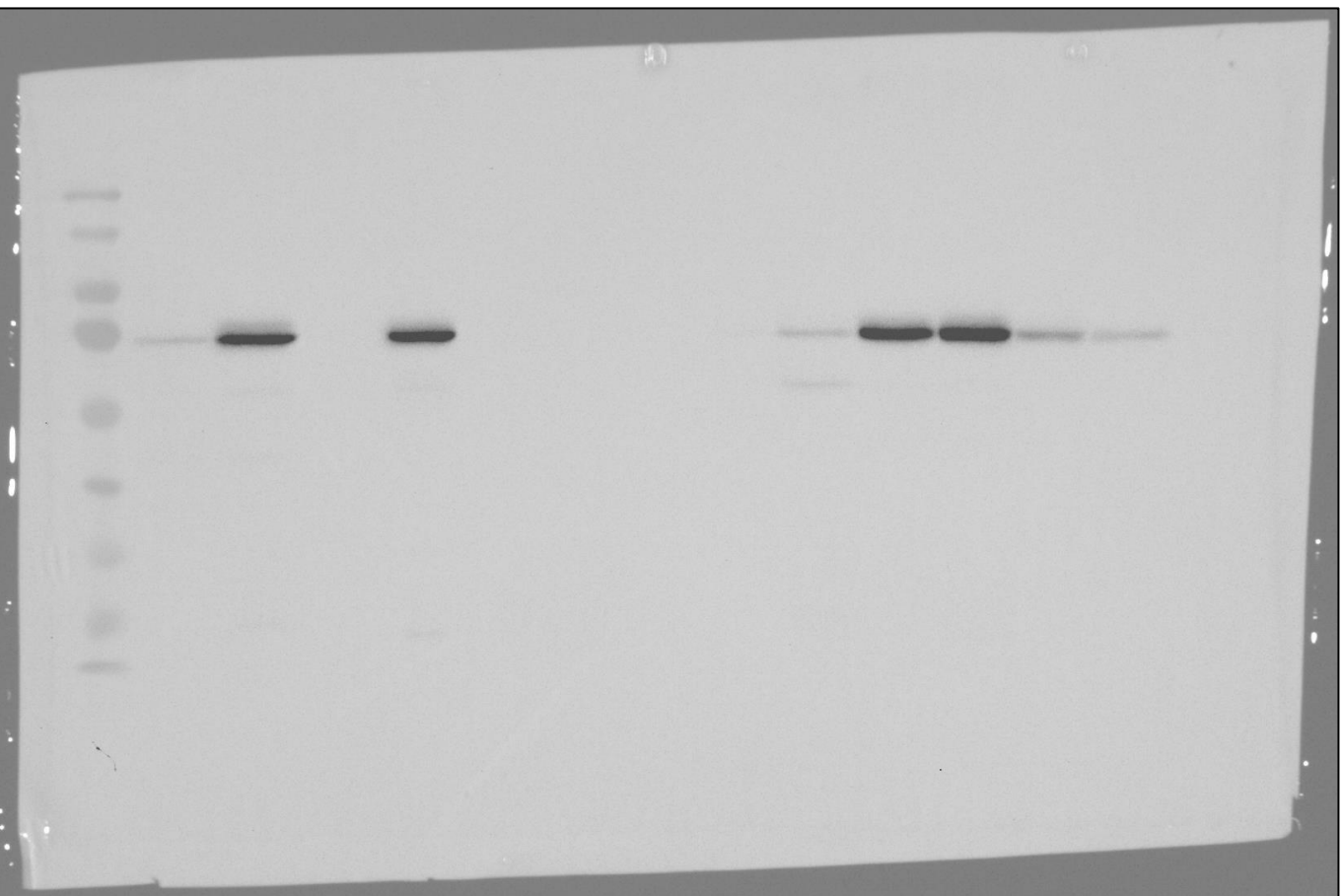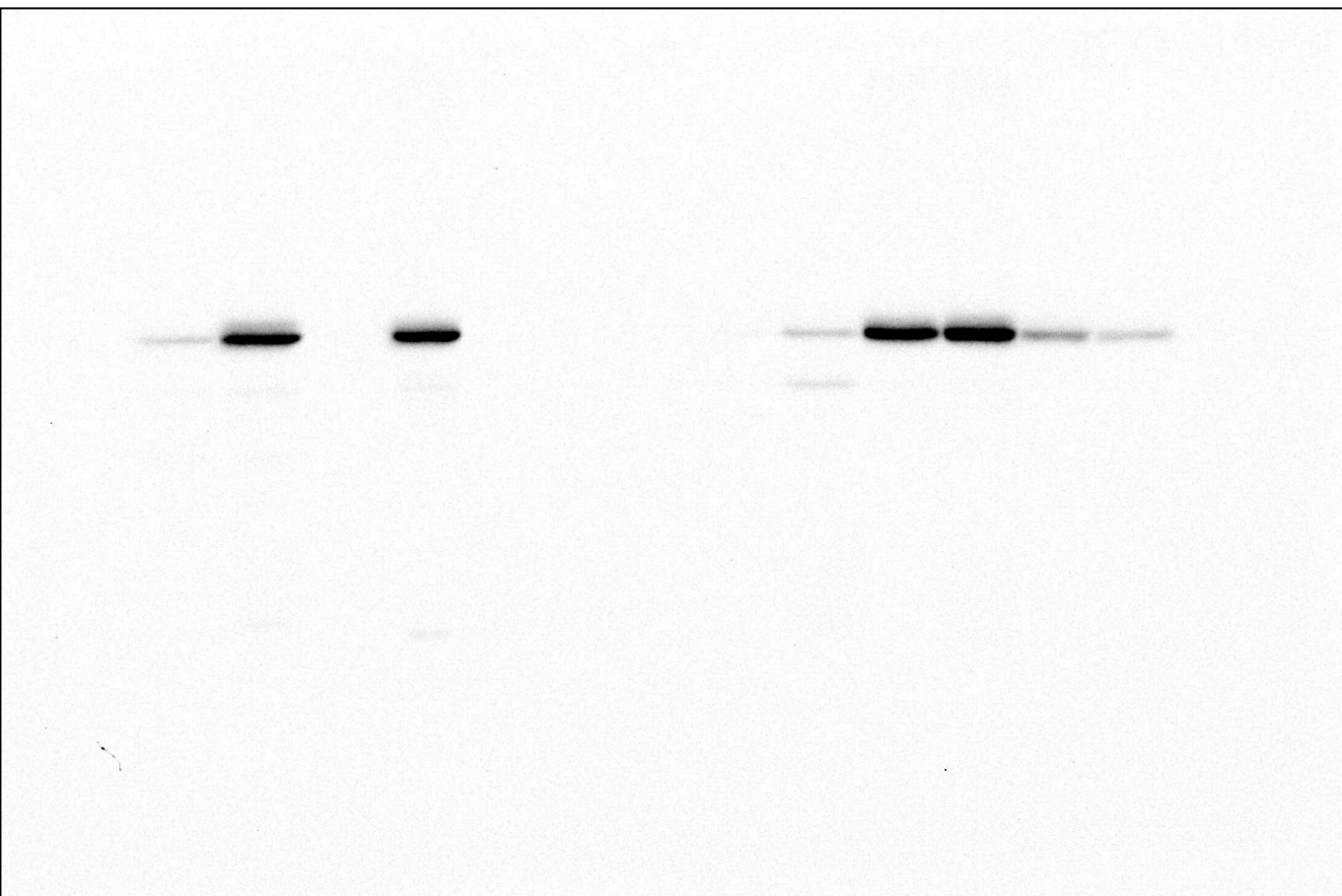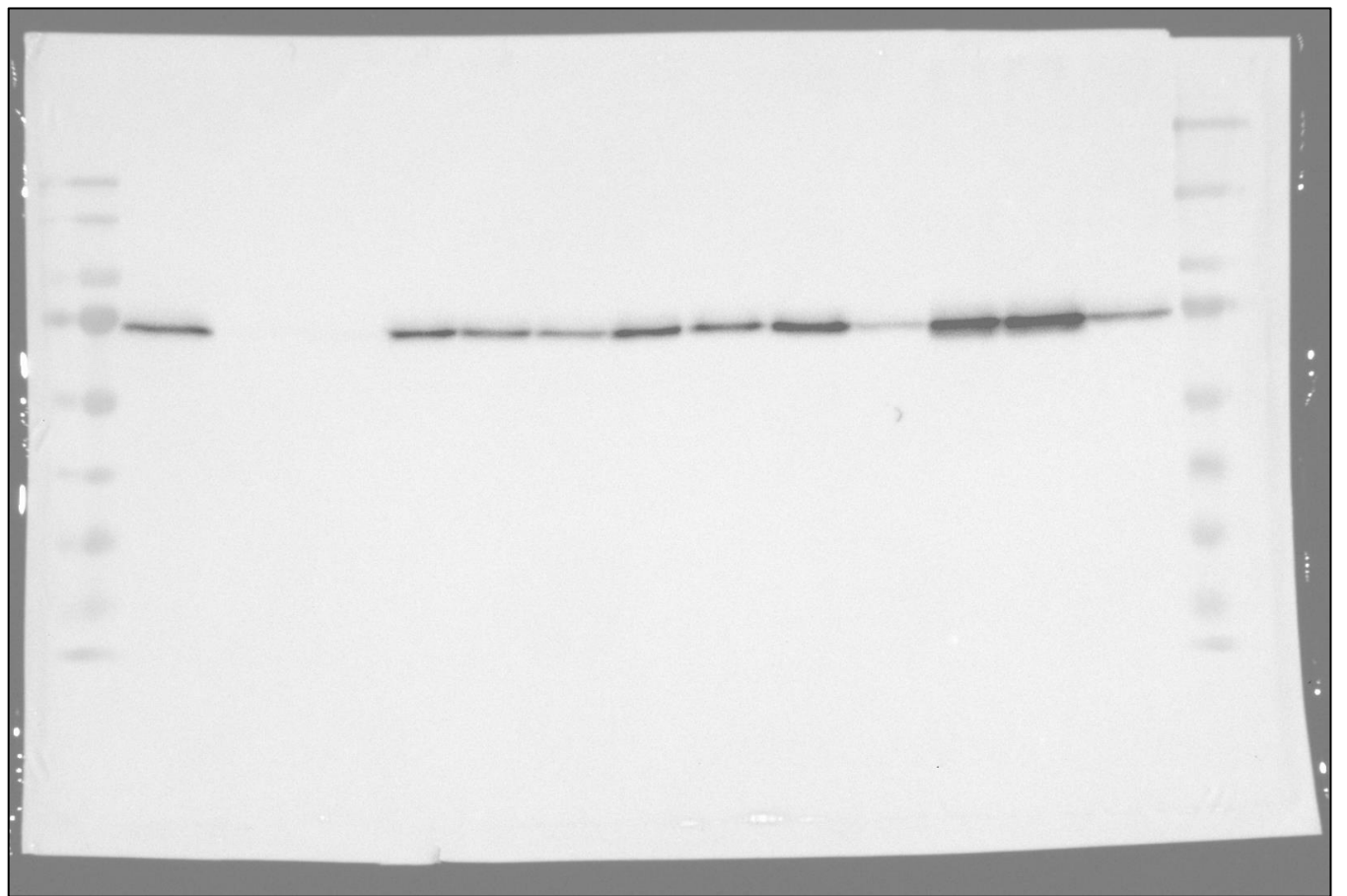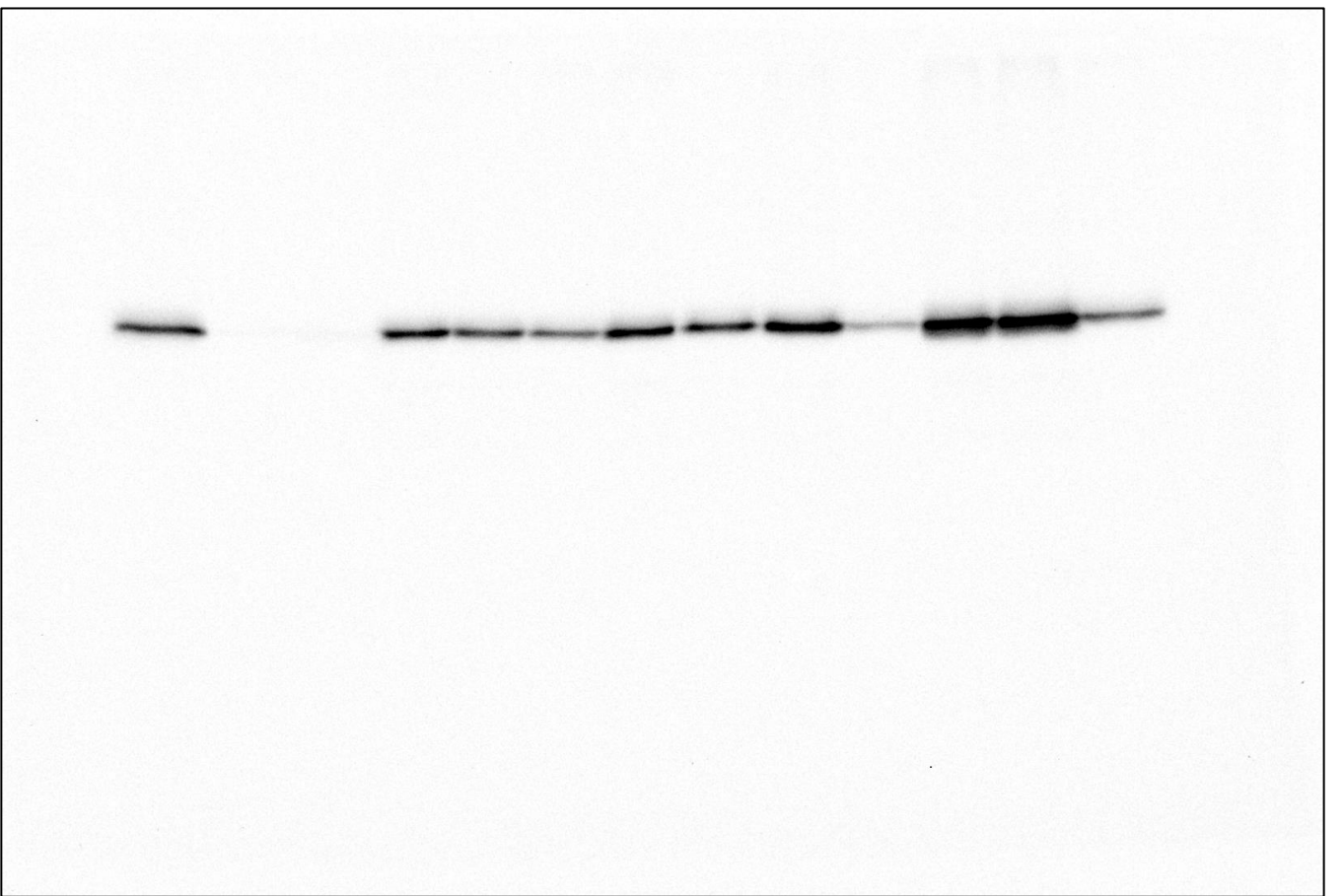

**IB: p-NFκB p65<sup>S536</sup>**  
65 kDa

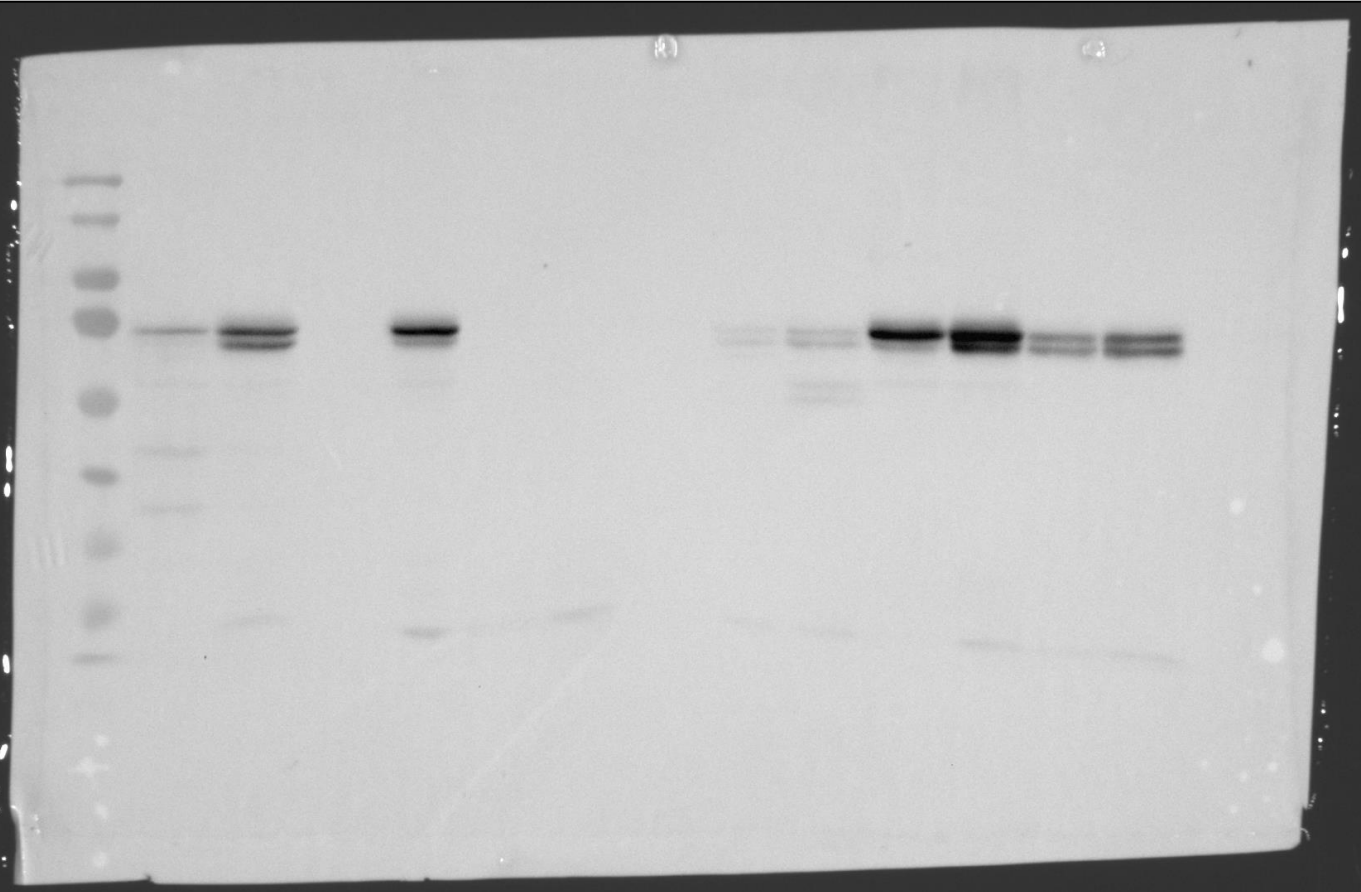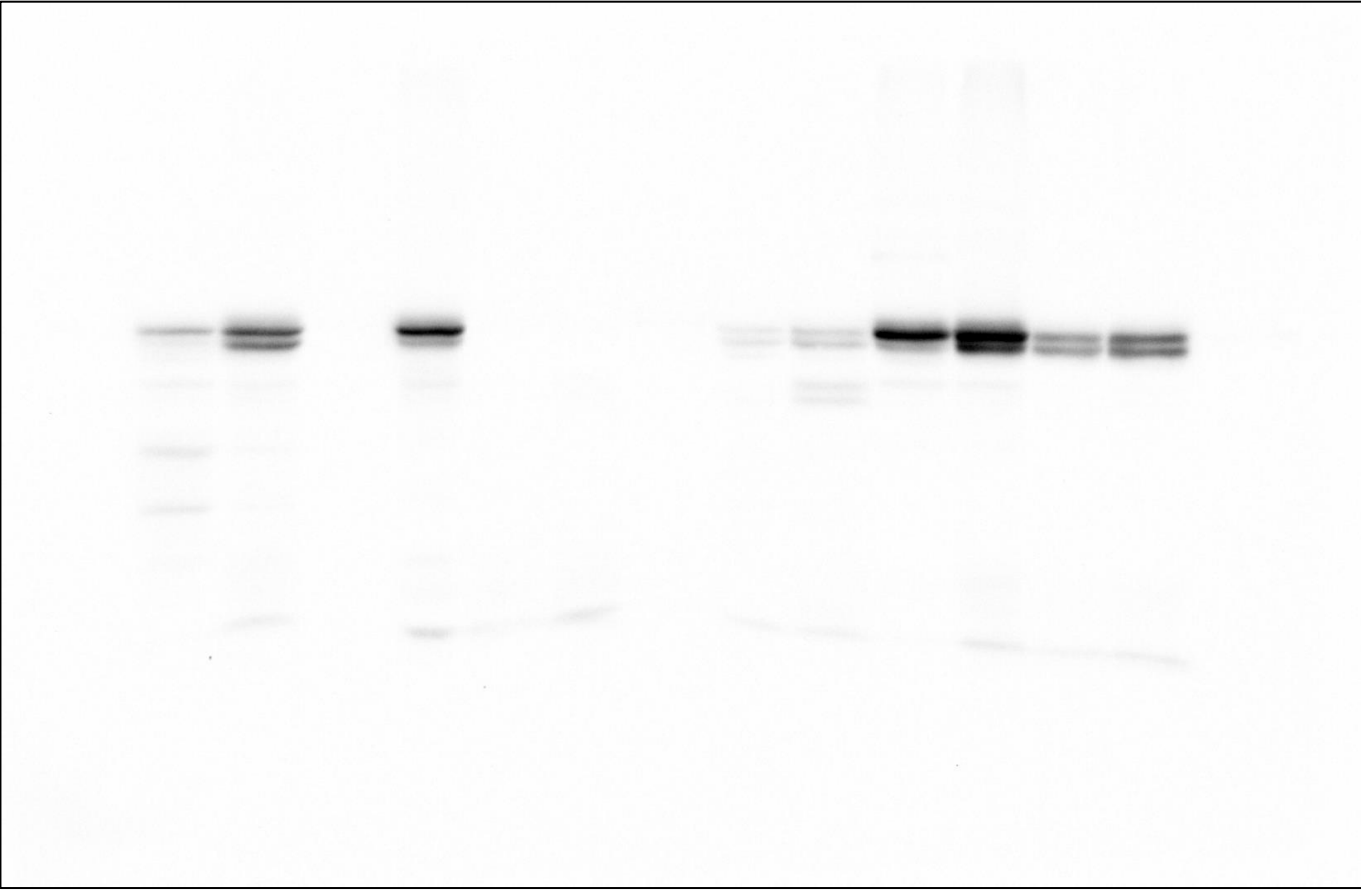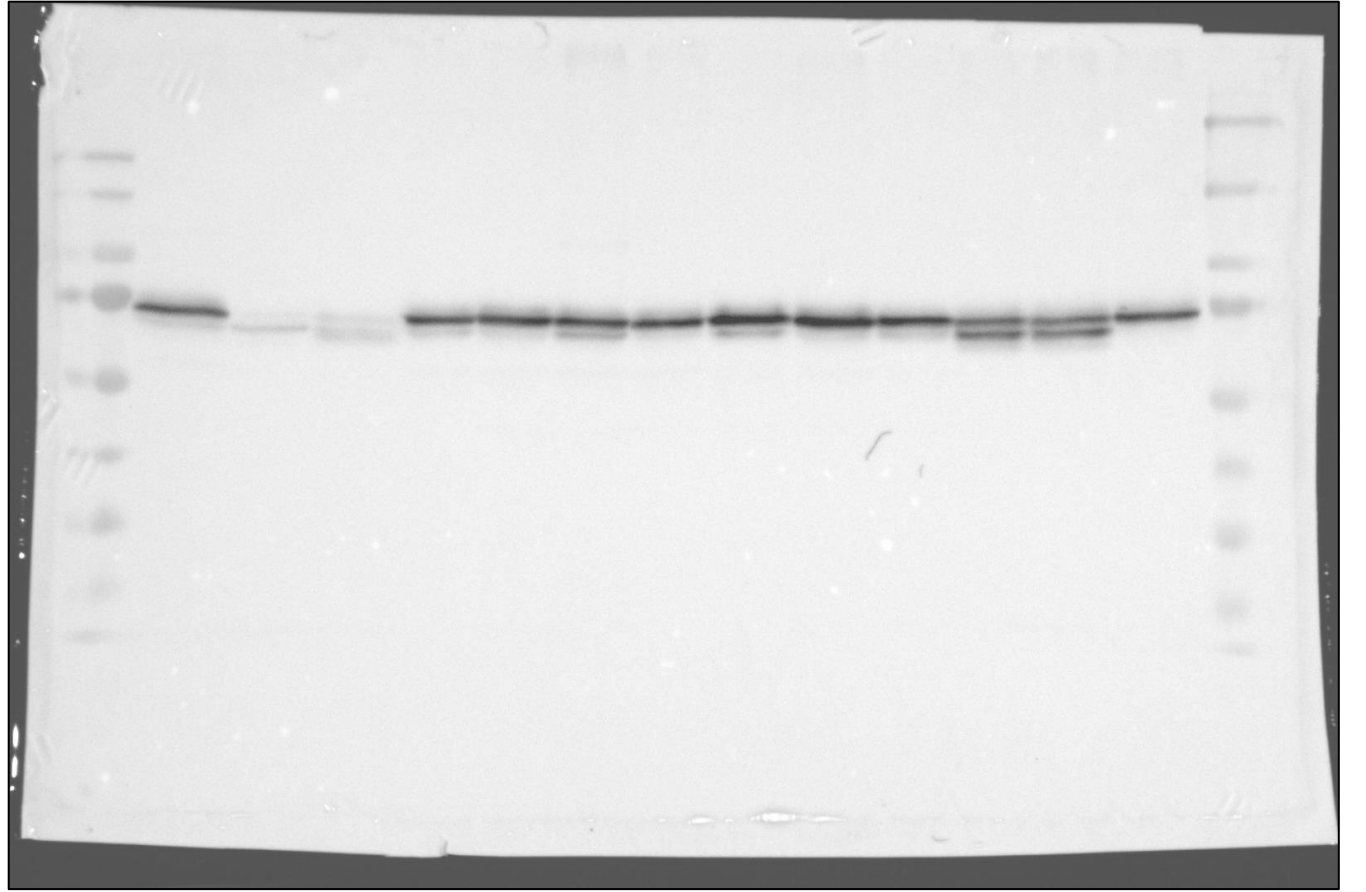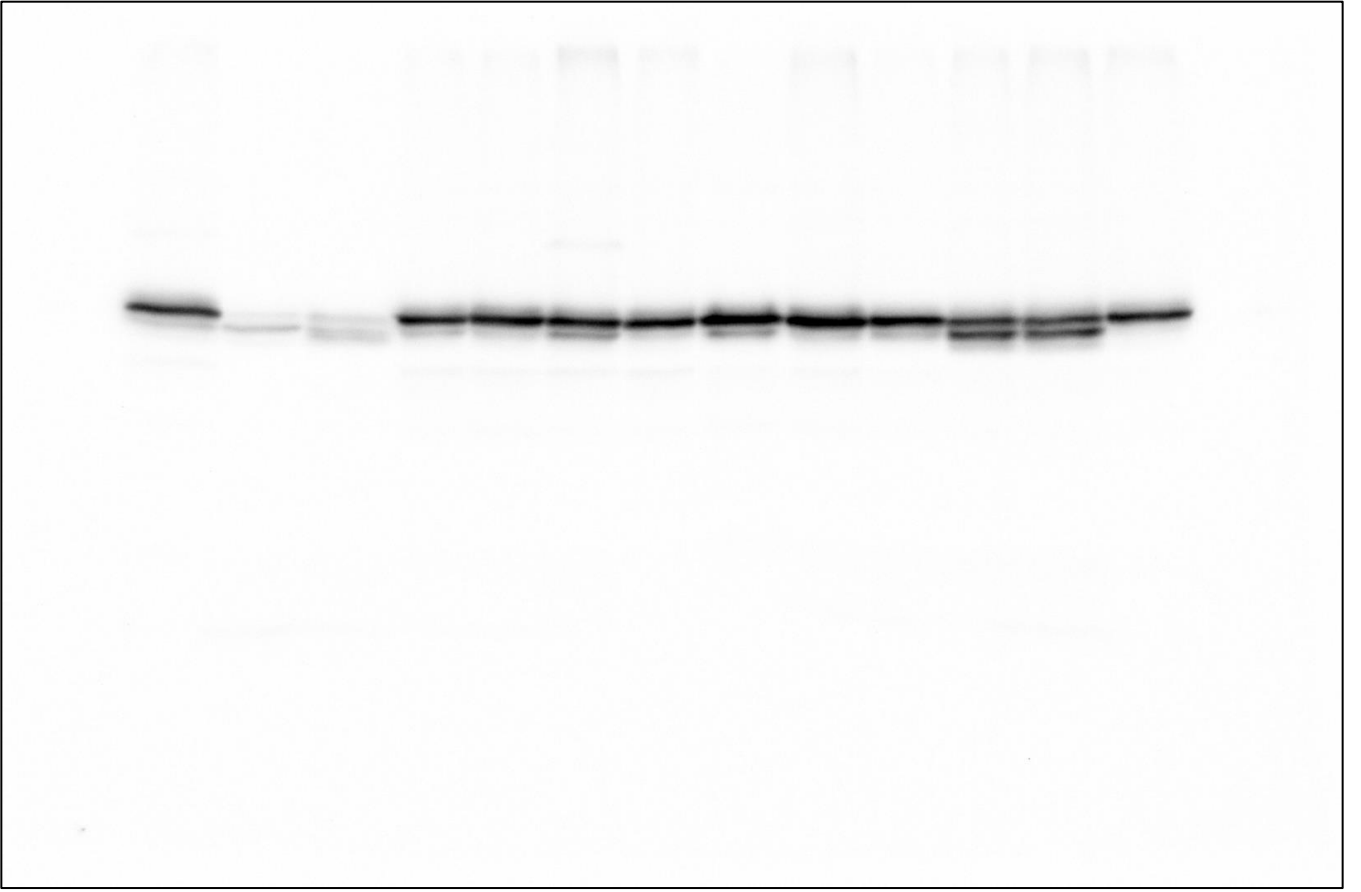

**IB: NFκB p65**  
65 kDa

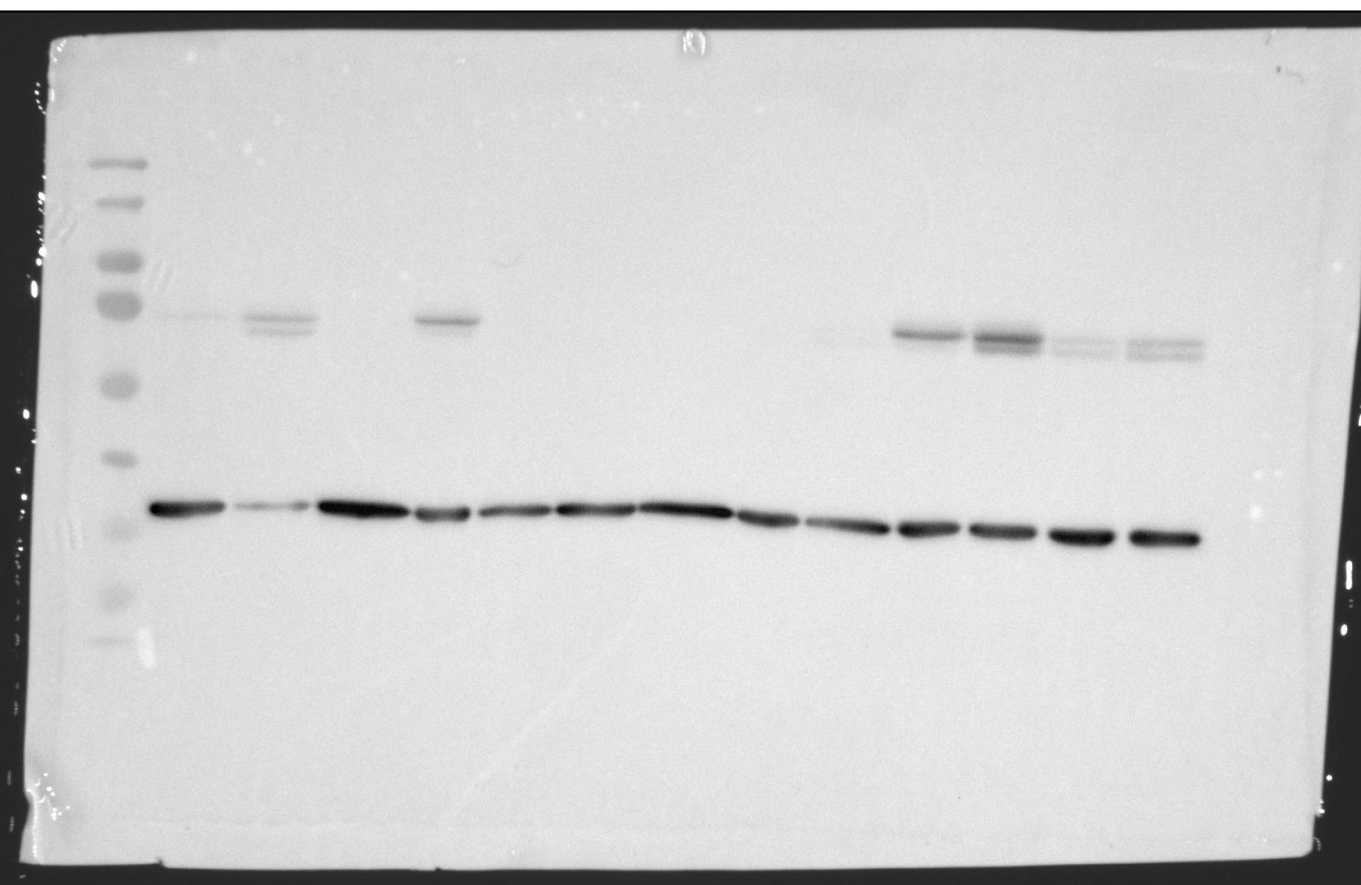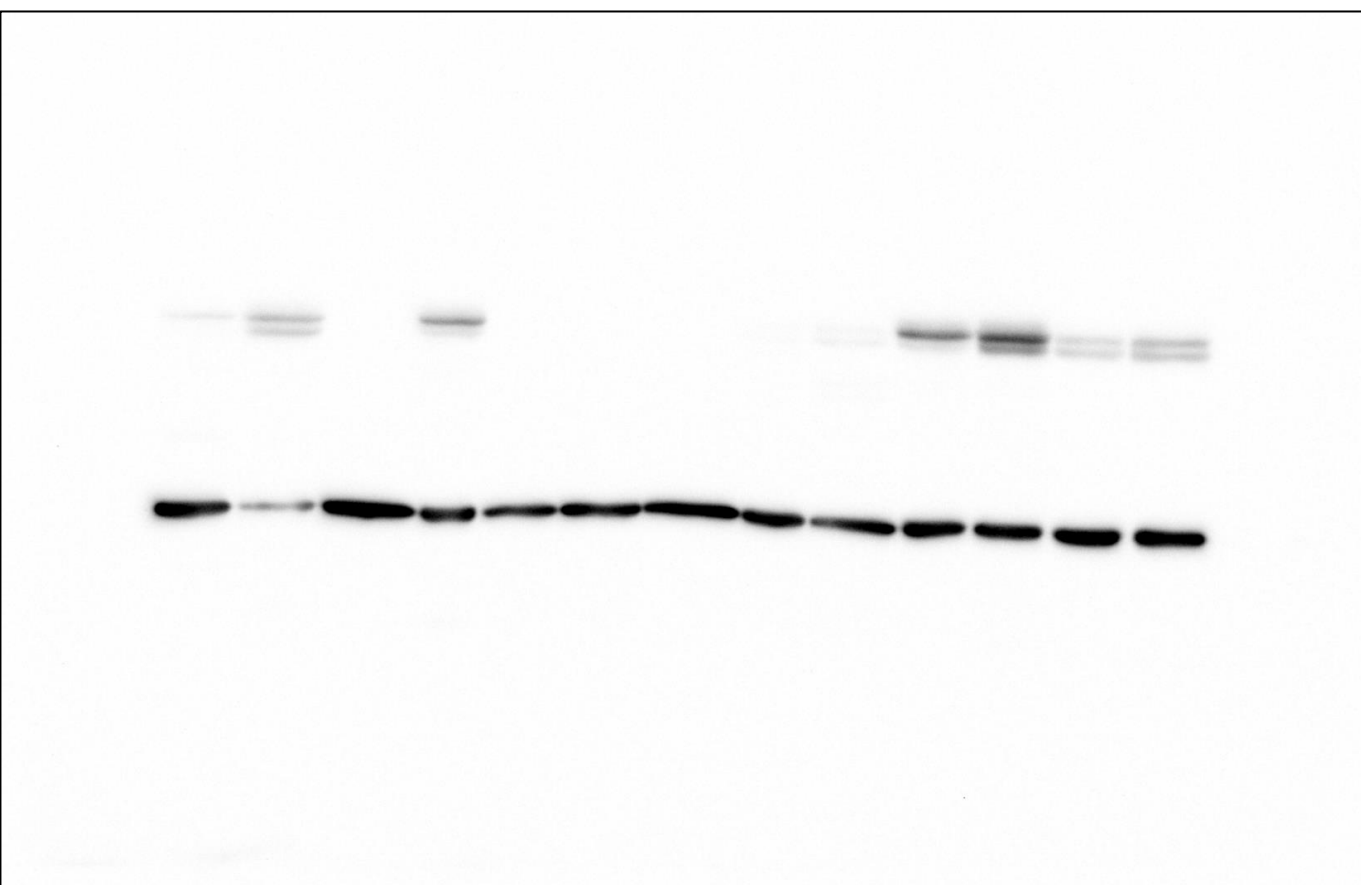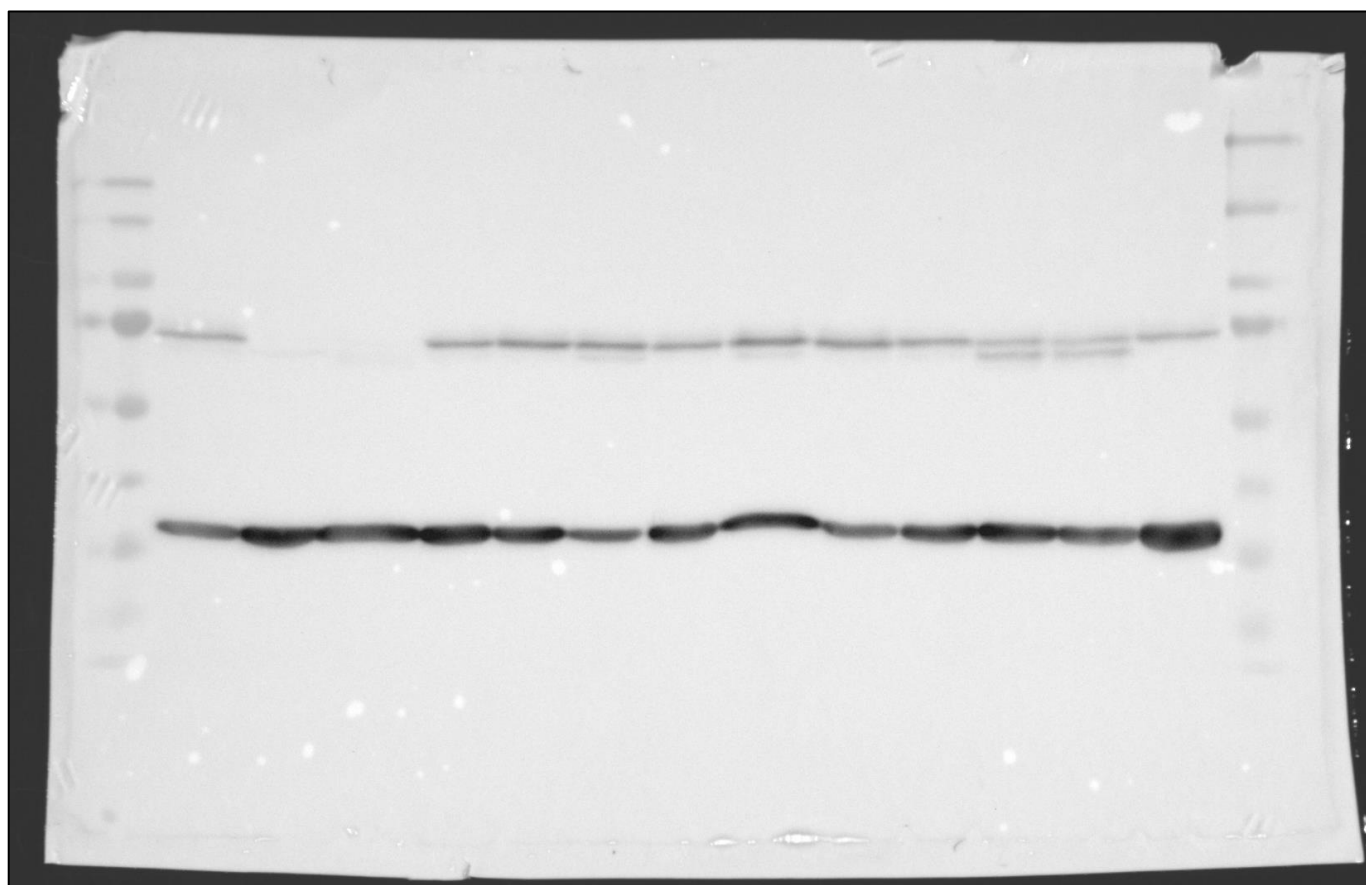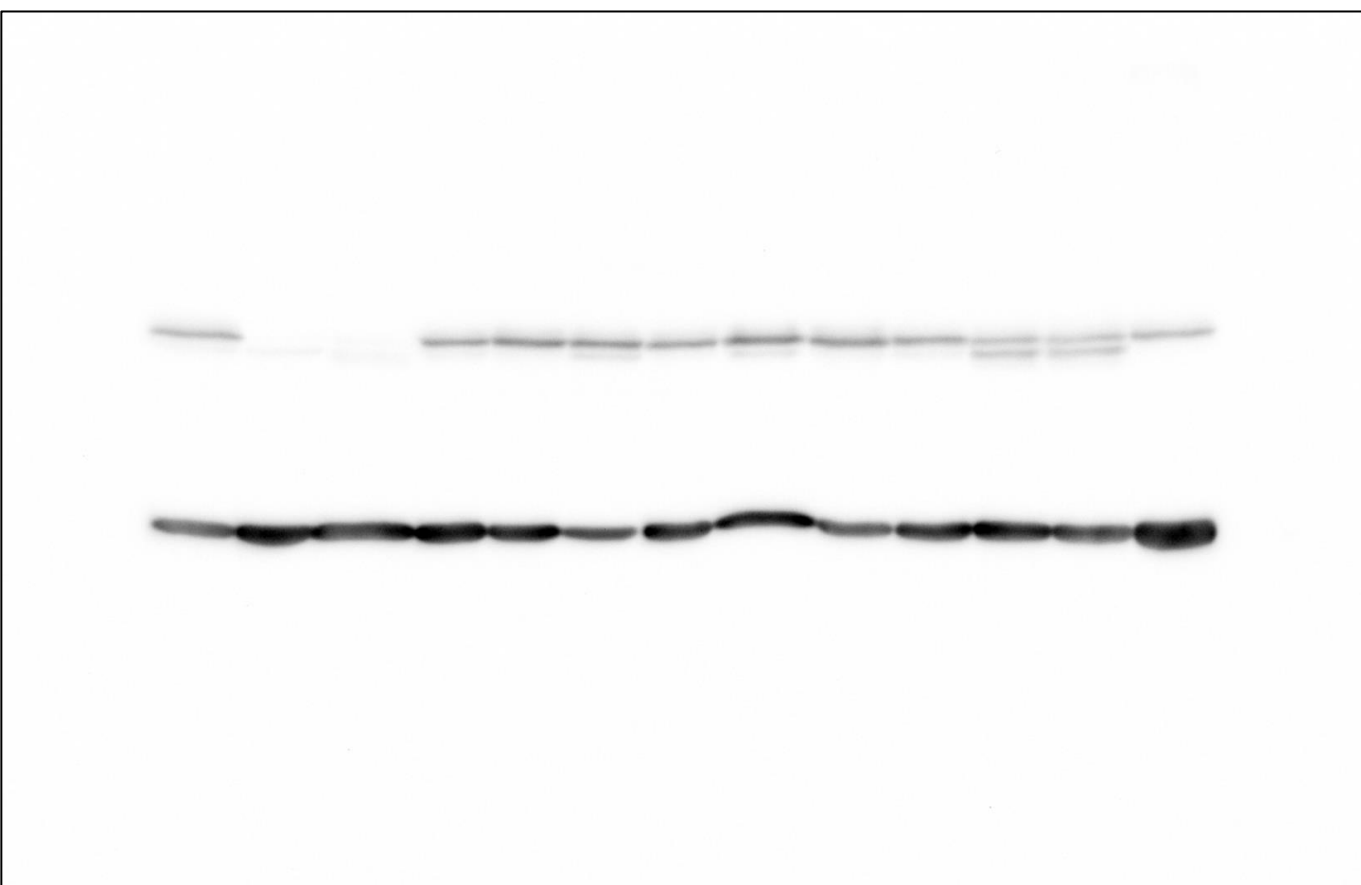

**IB: GAPDH**  
36 kDa

Supplement: Supplementary file 1 [file cancers-14-06080-s001.zip › Vicari et al_Figure S2.pdf]
